# A case of xanthogranulomatous orchitis and its preoperative diagnostic challenges

**DOI:** 10.1016/j.eucr.2020.101248

**Published:** 2020-05-15

**Authors:** Khaled A. Murshed, Noheir M. Taha, Mohamed Ben-Gashir

**Affiliations:** Department of Laboratory Medicine and Pathology, Hamad Medical Corporation, Doha, Qatar

**Keywords:** Xanthogranulomatous orchitis, Testis, Orchitis, Epididymo-orchitis

## Abstract

Xanthogranulomatous orchitis (XGO) is an extremely rare inflammatory disease of the testis which can mimic testicular tumors. We report a 42-year-old man who presented with left scrotal swelling for one-month duration associated with pus discharge from the overlying scrotal skin. Scrotal ultrasonography revealed an atrophic heterogenous left testis with scrotal wall collection. Surgical scrotal exploration with left simple orchidectomy was performed. By histopathology, the diagnosis of XGO was rendered. The preoperative diagnosis of XGO can very challenging and the diagnosis mainly relies on histopathologic examination. Adequate pathologic sampling is essential to rule out the possibility of co-existing testicular neoplasms.

## Introduction

Xanthogranulomatous inflammation is a well-recognized type of inflammation that can affect various organs in the body. In the genitourinary tract, it most commonly involves the kidney and urinary bladder. Its occurrence in the testis is a very rare event.^1^, ^2^, ^3^, ^4^, ^5^ To the best of our knowledge, up to 25 cases have been reported so far in the English literature. Herein, we report another case of xanthogranulomatous orchitis (XGO) and discuss its preoperative diagnostic challenges and the important role that histopathologic examination plays in reaching the correct diagnosis and exclusion of neoplastic process.

## Case presentation

**Fig. 1:**
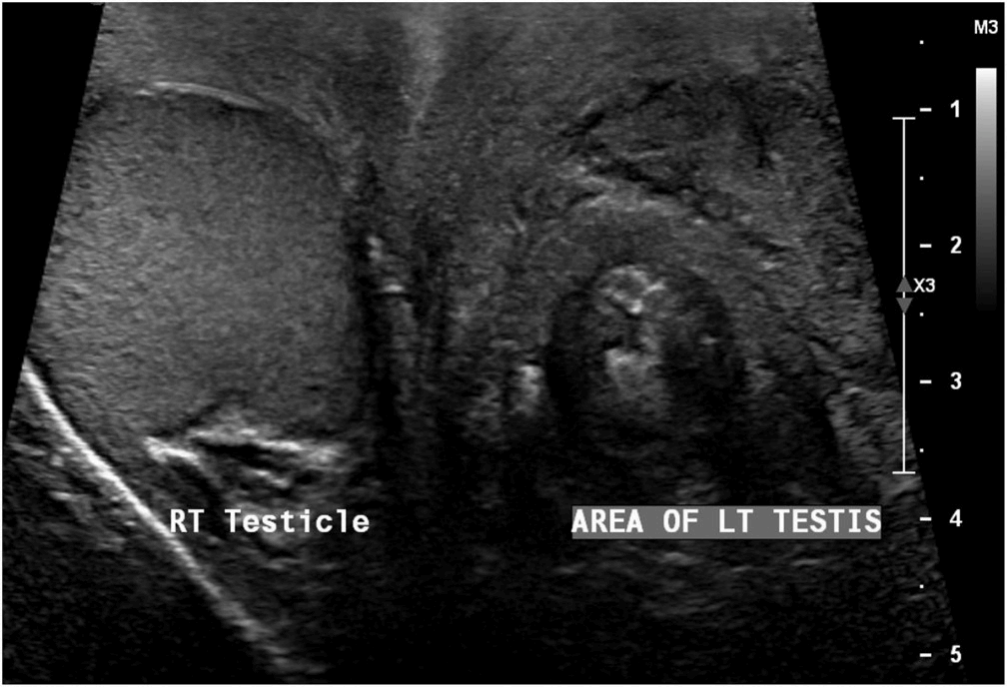
Ultrasound showing an atrophic left testis with heterogenous hyperechoic and hypoechoic areas along with collection in the scrotal wall.

**Fig. 2:**
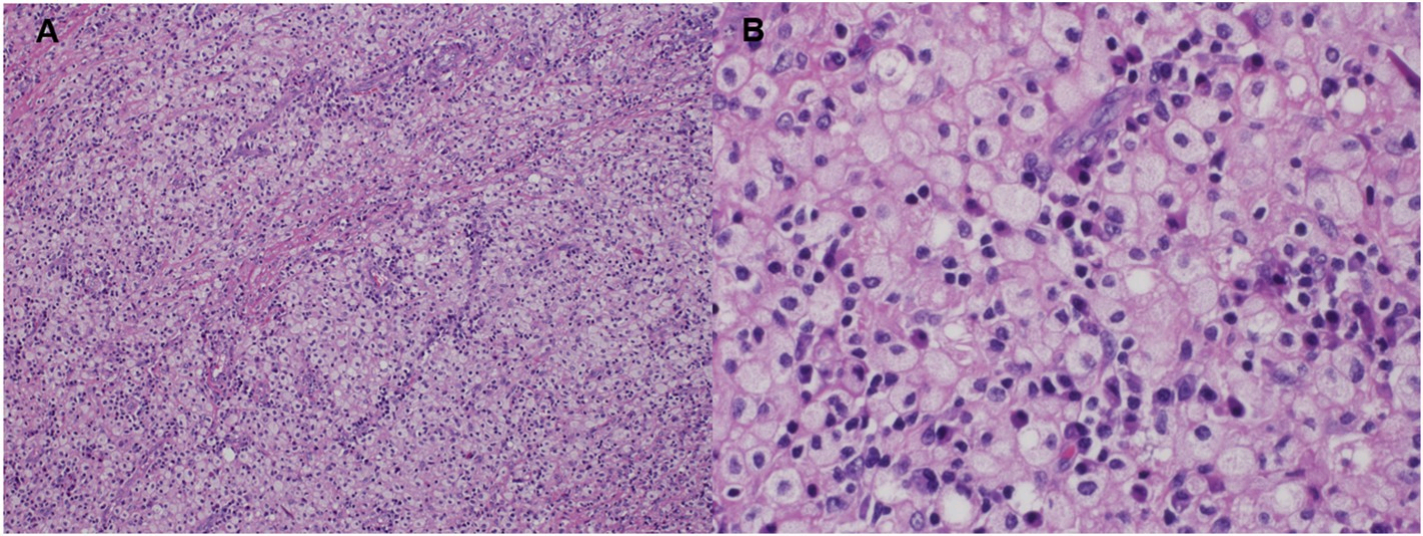
Microscopic features: **A,** photomicrograph showing xanthogranulomatous inflammation completely replacing the testicular parenchyma (Hematoxylin & Eosin stain, x100). **B**, high power view reveals foamy histiocytes intermixed with numerous plasma cells and lymphocytes (H&E stain, x400).

**Fig. 3:**
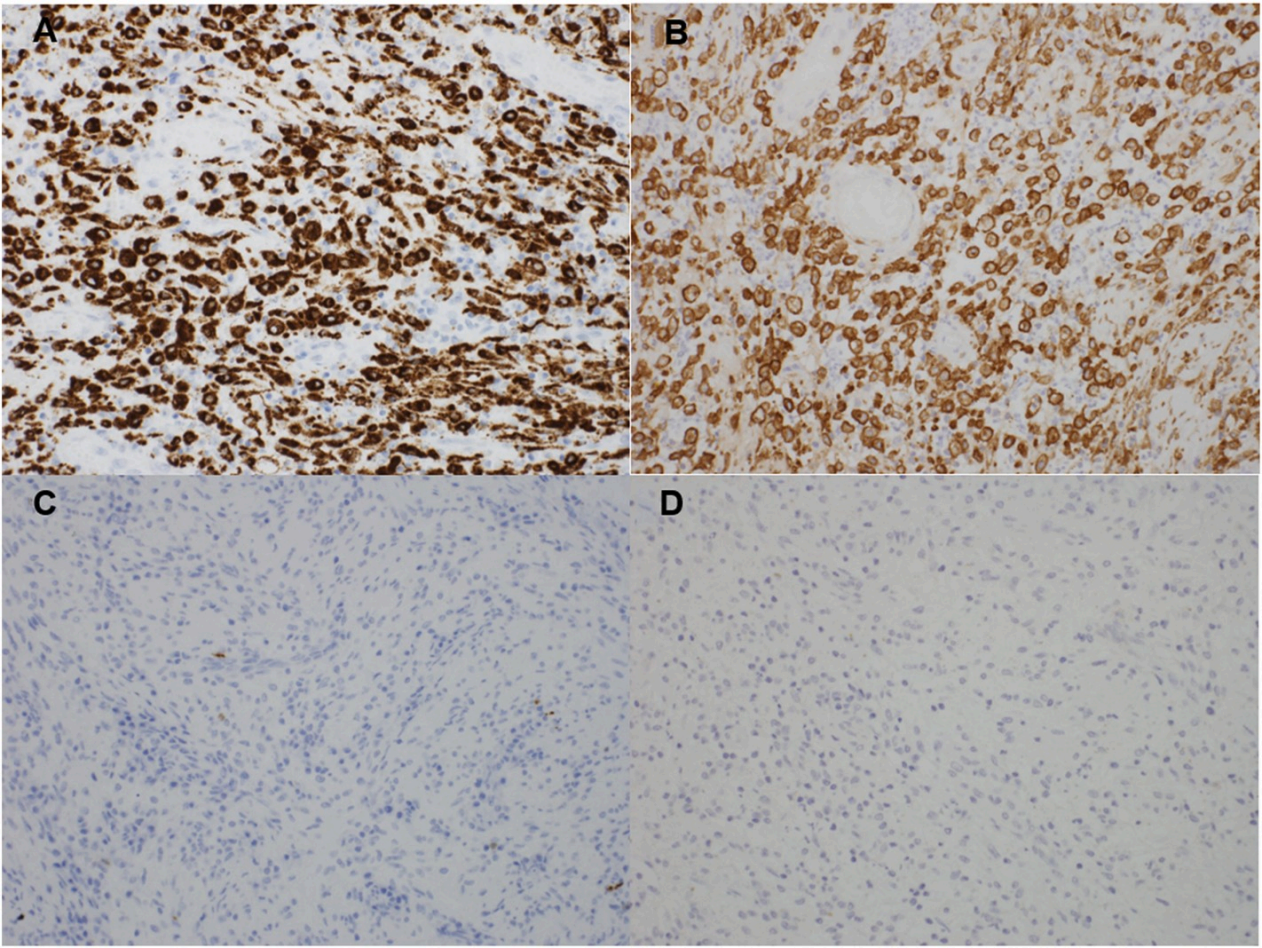
Immunohistochemical features: **A,** the foamy histiocytes are immunoreactive for CD68 antibody (immunohistochemistry, x200). **B,** CD163 is positive in the foamy histiocytes **(**IHC, x200). **C**, negative staining for S100. **(**IHC, x200) D, negative staining for CD1a. **(**IHC, x200).

A 42-year-old man presented with history of recurrent left sided scrotal swelling and dull pain for one-month duration. The scrotal swelling was associated with pus discharge from the anterior surface of the scrotum. Two weeks prior to that, he was seen for the same complaint and received antibiotics for two weeks with no response. The patient denied having urinary symptoms, urethral discharge, fever or any constitutional symptoms. He did not have any chronic illnesses, and he denied history of trauma or recent sexual contacts. Physical examination revealed left scrotal nontender swelling with overlying scrotal wall abscess. Urine culture was negative. Serum tumor markers were within normal range. Scrotal ultrasonography showed an atrophic heterogenous left testis with scrotal wall collection (Fig. 1). Surgical scrotal exploration was performed. A left simple orchidectomy along with drainage of the scrotal wall abscess was done. Histopathologic examination showed that the testicular parenchyma was diffusely replaced by proliferation of foamy histiocytes intermingled with lymphocytes, plasma cells and eosinophils, consistent with XGO (Fig. 2). Special stains for mycobacterial and fungal microorganisms were negative. The inflammatory process was focally extending into the epididymis and peritesticular soft tissue. showed that the foamy histiocytes are immunoreactive for CD68 and CD163 but negative for S100 and CD1a (Fig. 3). No evidence of neoplastic growth was identified in the entirely examined testis. The patient was discharged on analgesics and antibiotics. He is now on regular follow-up.

## Discussion

XGO is a rare non-neoplastic destructive inflammatory disease of the testis that can cause a mass-like lesion simulating malignancy.^5^ In other organs in the body that can be affected by this disease such as kidney, gallbladder and appendix, the etiology has been hypothesized to be related mainly to obstructive process and chronic infection.^2^, ^3^, ^4^ Likewise, in the testis, obstruction of the spermatic cord and urinary tract infection play a major role in pathogenesis. Infectious microorganisms most of the time cannot be detected by urine culture due to the chronic nature of the disease. The obstruction of spermatic cord can be either mechanical like in patients who underwent prostatectomy or transurethral prostate resection, or functional due to neurological disorders such as neuropathy that occurs in patients with diabetes mellitus, or as a result of spinal cord injury.^4^

The preoperative diagnosis of XGO can be challenging as the disease has similar clinical and radiological features to testicular neoplasms. Both conditions present with painless testicular swelling and can cause a mass-like lesion on radiological examination. Elevated serum tumor biomarkers can give a clue to diagnosis preoperatively. However, in some testicular neoplasms, serum tumor markers can be in normal range, which makes distinction between both conditions even more difficult and relies mainly on histopathologic examination of the resected specimen.

The identification of aggregates of foamy histiocytes intermingling with mixed inflammatory cell infiltrate destructing the testicular parenchyma is the typical microscopic finding in XGO. Histopathologic differential diagnosis mainly includes Malakoplakia, Rosai-Dorfman disease and infectious epididymo-orchitis.^3^^,^^4^ In our case, microscopic examination showed no features of Malakoplakia. Intracytoplasmic laminated concretions made of iron and calcium “Michaelis-Gutmann bodies” were not identified. The absence of emperipolesis (large histiocytes with pathognomonic lymphophagocytosis) along with negative staining of histiocytes for S100 immunostain, led to exclusion of Rosai-Dorfman disease. The lack of caseating granulomas with negative special stain for acid fast bacilli were against the diagnosis of tuberculosis. Finally, lepromatous orchitis was considered, but the negative special stains and lack of skin lesions were against this diagnosis.

## Conclusion

XGO is a rare inflammatory disease of the testis that can simulate testicular neoplasm. Careful macroscopic and microscopic histopathologic examination with adequate pathologic sampling are considered the gold standard for confirming this diagnosis and exclusion of neoplastic growth.

## Declaration of competing interest

The authors declared no potential conflicts of interest.
